# NS3 Protease from Hepatitis C Virus: Biophysical Studies on an Intrinsically Disordered Protein Domain

**DOI:** 10.3390/ijms140713282

**Published:** 2013-06-26

**Authors:** Sonia Vega, Jose L. Neira, Carlos Marcuello, Anabel Lostao, Olga Abian, Adrian Velazquez-Campoy

**Affiliations:** 1 Institute of Biocomputation and Physics of Complex Systems (BIFI), Joint Unit BIFI-IQFR (CSIC), University of Zaragoza, Zaragoza 50018, Spain; E-Mails: svega@bifi.es (S.V.); jlneira@umh.es (J.L.N.); 2 Institute of Molecular and Cell Biology, Miguel Hernandez University, Elche (Alicante) 03202, Spain; 3 Advanced Microscopy Laboratory (LMA), Institute of Nanoscience of Aragon (INA), University of Zaragoza, Zaragoza 50018, Spain; E-Mails: cmarcuel@unizar.es (C.M.); aglostao@unizar.es (A.L.); 4 ARAID Foundation, Government of Aragon, Zaragoza 50018, Spain; 5 IIS Aragon–Aragon Health Science Institute (I+CS), Zaragoza 50009, Spain; 6 Network Biomedical Research Center on Hepatic and Digestive Diseases (CIBERehd), Barcelona 08036, Spain; 7 Department of Biochemistry and Cellular and Molecular Biology, Faculty of Sciences, University of Zaragoza, Zaragoza 50009, Spain

**Keywords:** NS3 protease, protein folding and stability, ligand binding, conformational landscape, intrinsically disordered protein

## Abstract

The nonstructural protein 3 (NS3) from the hepatitis C virus (HCV) is responsible for processing the non-structural region of the viral precursor polyprotein in infected hepatic cells. NS3 protease activity, located at the *N*-terminal domain, is a zinc-dependent serine protease. A zinc ion, required for the hydrolytic activity, has been considered as a structural metal ion essential for the structural integrity of the protein. In addition, NS3 interacts with another cofactor, NS4A, an accessory viral protein that induces a conformational change enhancing the hydrolytic activity. Biophysical studies on the isolated protease domain, whose behavior is similar to that of the full-length protein (e.g., catalytic activity, allosteric mechanism and susceptibility to inhibitors), suggest that a considerable global conformational change in the protein is coupled to zinc binding. Zinc binding to NS3 protease can be considered as a folding event, an extreme case of induced-fit binding. Therefore, NS3 protease is an intrinsically (partially) disordered protein with a complex conformational landscape due to its inherent plasticity and to the interaction with its different effectors. Here we summarize the results from a detailed biophysical characterization of this enzyme and present new experimental data.

## 1. NS3 Protease from Hepatitis C Virus

**Figure 1:**
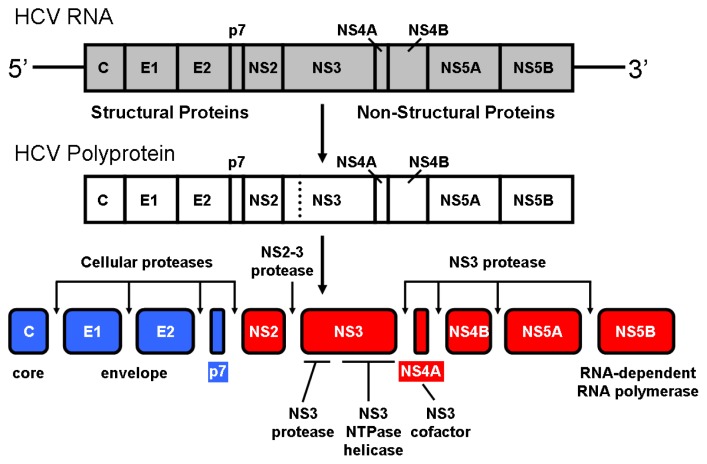
Processing of the hepatitis C virus polyprotein. Similar to other positive-strand RNA viruses, upon infection of a hepatic cell the genomic RNA of hepatitis C virus (9.6 kb, single-stranded) serves as messenger RNA for the translation of viral proteins. The linear molecule contains a single open reading frame coding for a precursor polyprotein (~3000 aminoacid residues) consisting of 10 proteins that must be cleaved in order to be functional.

The hepatitis C virus (HCV) infection is a worldwide health problem. HCV infected people amount to more than 200 million, with 80% of them becoming chronic patients, and many of them progressing to cirrhosis and hepatocellular carcinoma. Upon cell infection the viral RNA is directly translated into a precursor polyprotein which must be processed for successful viral maturation (Figure 1). Host cellular proteases are implicated in the processing of the structural viral proteins, whereas two different proteolytic activities encoded in the viral polyprotein, NS2 (or NS2/3) and NS3, are involved in the processing of the non-structural viral proteins. The NS2-NS3 junction is cleaved intramolecularly by the NS2/3 protease activity. This protein comprises the NS2 *C*-terminal and the NS3 *N*-terminal domains and is a zinc-dependent protease (metallo- or cysteine-protease) [1–4]. The remaining sites (NS3-NS4A to NS5A-NS5B) are cleaved by the NS3 protease, which is a zinc-dependent serine protease comprising the NS3 *N*-terminal domain [5–8].

Since its identification, the NS3 protease active site has been considered a pharmacological target for drug discovery. Although very few protease competitive inhibitors have successfully entered clinical trials, a broad variety of competitive inhibitors has been developed. Very recently, two protease inhibitors have been approved by the Food and Drug Administration for therapeutic treatment [9,10]. However, new antiviral agents are urgently needed because resistance mutations causing an efficacy reduction for these two drugs have already been identified [11–13]. Moreover, the NS4A binding site constitutes another weak point in the NS3 protease, which has also been considered a drug target for allosteric inhibitors: a small molecule blocking the NS4A binding site could hinder the conformational change elicited by NS4A binding required for enhancing NS3 activity. In addition, the zinc binding site has also been hypothesized to be a valid target for drug design [14]. For this goal, understanding the energetics of the NS3-zinc interaction and its impact on the structural stability of the enzyme may be valuable in getting insight into the design and development of possible drugs against the metal binding site. The biophysical and functional characterization of a pharmacological protein target is important because its conformational behaviour may provide important information on its function, on potential weak points for small-molecule ligand design, on the impact of resistance-associated mutations in its conformational stability, or on new methodologies for identifying bioactive ligands.

In this article we will review two interrelated aspects of the NS3 protease:

(1) The energetics of zinc binding to NS3 and its structural implications [15]. Different biophysical techniques have been employed for probing the structural features of the zinc-bound and the zinc-free NS3 conformations. In particular, spectroscopy data (circular dichroism, fluorescence and ^1^H-1D-NMR) suggested a significant conformational change (in tertiary structure, mainly) associated with zinc interaction [15]. Furthermore, isothermal titration calorimetry (ITC) has been used for determining the energetics of the NS3-zinc interaction, confirming a remarkable conformational change coupled to zinc binding from the determination of the binding heat capacity [15]. Because ITC provides a direct measurement of the binding enthalpy, it is possible to get a reliable estimation of the change in heat capacity upon binding, which is very difficult or simply infeasible using spectroscopic techniques. In addition, here we report translational diffusion experiments and atomic force microscopy data providing further information on the zinc-dependent plasticity of the NS3 protease.
(2) A description of the conformational landscape of NS3. The structural molecular integrity is dominated by the interaction with zinc. The binding of zinc induces a significant structural global rearrangement of the protein: spectroscopic and calorimetric thermal denaturation assays indicate that zinc-free NS3 protease exhibits very low stability, whereas the zinc-bound NS3 protease is considerably stabilized due to metal binding [16]. Contrary to other homologous zinc-dependent proteases, the zinc-free NS3 protease is not completely unstructured. Thus, the NS3 protease exhibits a fairly complex conformational equilibrium.

## 2. NS3 Protease: Structure and Function

**Figure 2:**
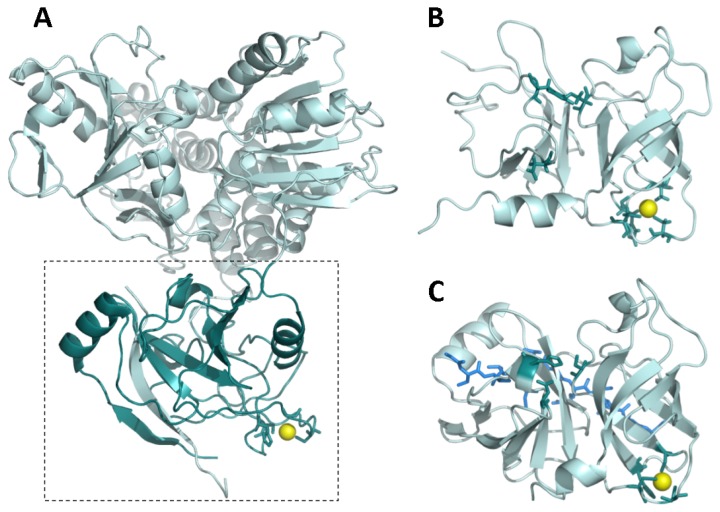
(**A**) Full-length NS3 protein from hepatitis C virus (PDB code: 1CU1) [26]. The dashed rectangle delimitates the protease domain (dark cyan). The zinc ion is shown as a yellow sphere. The NS4A cofactor-mimicking peptide incorporates into the NS3 protease domain as an additional beta strand (cyan); (**B**) NS3 protease domain in the absence of NS4A cofactor (PDB code: 1BT7) [27]. The zinc ion is shown as a yellow sphere. The zinc-coordinating residues and the catalytic residues are shown as dark cyan sticks; (**C**) NS3 protease domain in the presence of a NS4A cofactor-mimicking peptide (blue sticks) (PDB code: 1JXP) [28]. The zinc ion is shown as a yellow sphere. The zinc-coordinating residues and the catalytic residues are shown as dark cyan sticks. The NS4A cofactor-mimicking peptide incorporates into the NS3 protease domain as an additional beta strand (blue sticks). Comparison between (**B**) and (**C**) reveals a structural rearrangement affecting the *N*-terminal domain of NS3 protease upon NS4A binding and propagating to the catalytic triad (for example, D81 is reoriented upward, towards a productive conformation).

Most enzymatic and biophysical studies on NS3 protease have been performed with the isolated *N*-terminal domain from full-length NS3 protein, which exhibits similar properties (enzymatic activity, inhibition constants, and allosteric activation mechanism) to those of the full-length protein [17]. NS3 protease is a serine protease structurally homologous to other extracellular serine proteases, like trypsin and chymotrypsin (Figure 2). The secondary structure is dominated by β-strands and turns, confirmed by its far-UV circular dichroism spectrum. The catalytic active site is located in a groove between the *N*- and *C*-termini. Homologous extracellular proteases present disulfide bridges stabilizing the molecular structure. However, as expected for an intracellular protease working under reducing conditions, NS3 does not contain disulfide bridges, but a zinc ion tetra-coordinated by three cysteine residues and a histidine residue in its *C*-terminal domain [6]. The zinc ion is required for the hydrolytic activity, since its removal leads to inactivation, but it is located very far (>20 Å) from the catalytic His-Asp-Ser triad (H57/D81/S139 in NS3 numbering) to be involved in catalysis. Consequently, the zinc ion is considered to have a structural role, stabilizing the protein active conformation. In fact, the NS3 zinc binding site contains the characteristic CXC...CXXXH signature for structural zinc binding sites (C97/C99/C145/H149 in NS3 numbering) [5,14,18]. Thus, the zinc ion is supposed to be structurally equivalent to the disulfide bridges found in other serine proteases, and it may be involved in the folding or the post-folding stability of the enzyme, or both. In addition, any perturbation at the zinc binding site may propagate to the active site, since both regions are linked by two β-strands.

It has been suggested that both protease activities, NS2/3 and NS3, rely on the same zinc ion bound to NS3, but with the zinc ion having a catalytic role in NS2/3 protease and a structural role in NS3 protease [1,8,19,20]. Furthermore, the three zinc-coordinating cysteines in NS3 (C97/C99/C145) are required for both NS2/3 and NS3 hydrolytic activities; however, another cysteine residue in NS2 is required for NS2/3 activity, whereas the histidine residue in NS3 is not needed [18]. In addition, the NS3 *N*-terminal domain, but not its activity, is required for NS2/3 activity, and the NS2/3 activity was shown to be more sensitive than the NS3 activity to inhibition by zinc-chelating agents [19]. Therefore, the zinc bound to NS2/3 and NS3 proteases may be the same metal ion, bound to structurally different binding sites sharing common residues, playing a catalytic and structural role in NS2/3 and NS3 proteases, respectively. A conformational change may occur in NS3 upon intramolecular cleavage of the NS2-NS3 junction, leading to alterations in the zinc coordinating cage (C...CXC...C to CXC...CXXXH) and increasing the zinc binding affinity [19].

In addition to the zinc interaction, NS3 protease also requires the binding of the viral protein NS4A [21–24], which provides: (1) additional structural stabilization, restructuring the *N*-terminal domain of NS3 protease; (2) enhancement of the proteolytic activity, changing the configuration of the catalytic triad of NS3 protease; and (3) appropriate cellular membrane localization, through a highly hydrophobic terminal fragment. In addition, it has been shown that: (1) NS3 is not completely inactive in the absence of NS4A, exhibiting a significant non-zero basal proteolytic activity, in particular in some of the cleavage sites; (2) the binding of NS4A has an effect on the zinc binding site, and, therefore, there must be some cooperative interaction in the binding of both NS4A and zinc ion [14]; and (3) NS4A binding to NS3 is weak and strongly dependent on solvent composition, requiring co-solutes (glycerol and detergents) mimicking the intracellular and near-membrane environment [25].

## 3. NS3 Protease: Zinc Interaction

Metals perform a variety of tasks in cells. In particular, zinc plays essential roles as cofactor of metabolic enzymes and transcription factors, and it is one of the most commonly bound transition metals in proteins, second only to iron [29]. Zinc is well-suited for its role in protein structure because: (1) it is not redox-active; and (2) it is relatively labile, exchanging very fast during ligand exchange reactions. Zinc binding sites in proteins can be classified as: (1) sites which mainly play a catalytic role; (2) those which have a regulatory role; and (3) sites that have a structural role. The effects of structural zinc ions in proteins may range from folding completely a natively-unfolded protein [30] to changes in protein stability [31].

According to a recent report structural and catalytic zinc binding sites in proteins can be distinguished by the ligand preference [18]: structural zinc binding sites are coordinated by at least two cysteine residues and no water molecules, whereas catalytic zinc binding sites are coordinated by at least two histidine residues and a single water molecule. In NS3 protease the zinc binding site is tetrahedrally coordinated by three cysteine residues and a histidine, where the interaction with the histidine is labile and can be replaced by a water molecule [6]. Therefore, the NS3 zinc binding site is an exception to the ligand preference rule for classifying zinc binding sites, because it exhibits features from both groups. The NS3 zinc binding site might correspond to both groups, as it has been reported that the ion is required for both NS2/3 and NS3 proteolytic activities with catalytic and structural functions, respectively, made possible by different protein conformations and zinc coordinating cages. The versatility of the zinc binding site in NS3 is apparent when comparing to other similar extracellular serine proteases. The stabilizing role of several disulfide bonds, strong covalent bonds, in chymotrypsin or trypsin, is substituted by just one non-covalent protein-cation interaction in NS3 protease. This is an indication of the expected strength and importance of such interaction.

At the same time, the homology between the structural roles of the zinc ion and the disulfide bonds in serine proteases leads to a significant difference between intracellular and extracellular serine proteases regulation. NS3 protease is an intracellular enzyme and the presence of a bound zinc ion required for its hydrolytic activity is going to be regulated by the internal free zinc pool inside the cells. There is some controversy about the concentration of free zinc within the cell, but normal levels are in the low nanomolar range [32]. As soon as the internal concentration of free zinc slightly increases, regulatory processes are initiated in order to remove any excess of zinc [33–35]. The accepted hypothesis establishes that cells do not operate with any significant pool of free zinc and that zinc homeostasis and occupancy processes in the cell are under kinetic control [36]. Following this idea, it has been shown that, similarly to most zinc enzymes, the interaction of NS3 protease with zinc exhibits a very slow kinetics, with small kinetic association and dissociation constants [14].

At pH 5, the binding of zinc to NS3 protease is entropically driven, with an opposing binding enthalpy, and the strength of the interaction is moderate (dissociation constant ~0.5 μM) [15]. The binding affinity of the NS3-zinc interaction at neutral pH must be much higher, because, contrary to what happens at pH 5, EDTA is not able to remove zinc from the enzyme [5]. This pH dependency of the binding indicates that there is a proton exchange (in this case, a net release of protons from the complex to the bulk solution) coupled to the binding process, in which probably the zinc-coordinating histidine is taking part.

**Figure 3:**
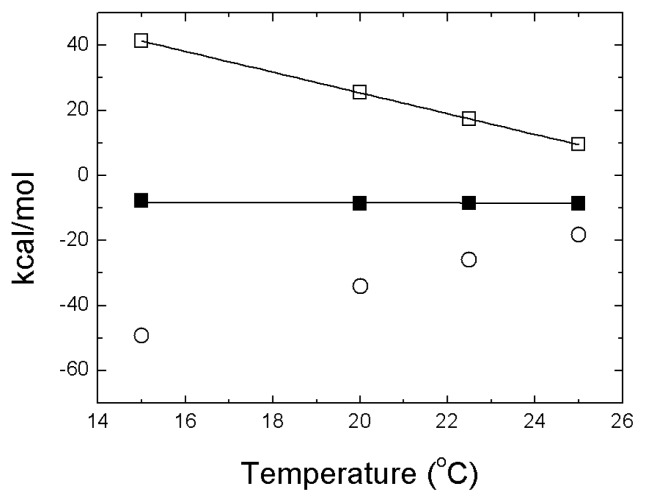
Thermodynamic dissection of the NS3 protease-zinc interaction. Temperature dependence of the Gibbs energy (ΔG, closed squares), enthalpy (ΔH, open squares) and entropy (−TΔS, open circles) for zinc binding to NS3 protease determined by ITC at pH 5. The lines correspond to the global non-linear regression fits for the temperature dependency of the Gibbs energy and enthalpy of interaction, considering a constant binding heat capacity. The strong temperature dependencies of enthalpy and entropy of binding suggest a considerable structural rearrangement coupled to metal ion binding.

The most outstanding feature of zinc binding to NS3 protease is the strong temperature dependence of the binding enthalpy [15]. The Gibbs energy of binding shows negligible temperature dependence, while both enthalpic and entropic contributions are strongly dependent on temperature, a nice example of enthalpy-entropy compensation (Figure 3). The estimated change in heat capacity upon binding of −3.2 kcal/K·mol is equivalent to the folding of an average protein of 20 kDa (the molecular mass of NS3 protease itself) [37].

Spectroscopic data (near-UV circular dichroism and fluorescence spectra) in the absence and presence of zinc bound to NS3 protease also suggest a considerable conformational change upon zinc binding involving mainly the weakening and/or distortion of the tertiary structure of the protein (since the far-UV circular dichroism is hardly affected by the presence of zinc) [15]. In addition, ANS-binding experiments and 1D-NMR spectra also suggest a global conformational change accompanied by solvent-exposure of hydrophobic regions upon zinc removal [15]. Interestingly, the hydrodynamic radius of NS3 protease only slightly increases (about 7%) when zinc is removed, according to DOSY-NMR measurements (see below).

Because the zinc-NS3 interaction enthalpy is positive, zinc binding is entropically driven. It should be borne in mind that when a conformational change is coupled to ligand binding, the global binding parameters contain contributions from the intrinsic protein–ligand interaction and the conformational change accompanying the binding process. Thus, the thermodynamic profile (ΔG, ΔH and −TΔS) for the global binding process is a combination of the thermodynamic profiles for the intrinsic binding process and the associated conformational change [15,38,39]. Considering that the binding event is coupled to a structuring process (gain of structure in the protein), the unfavorable conformational entropy loss due to the folding process elicited by binding, together with the roto-translational entropy loss characteristic of any binding process, must be compensated by a highly favorable desolvation entropy gain. Regarding the enthalpy, the intrinsic zinc binding enthalpy and the enthalpic contribution from the folding process, give rise to the unfavorable positive global binding enthalpy.

The binding enthalpy exhibits a linear dependence on temperature. Although this kind of enthalpy dependence is usually considered as an evidence for the absence of conformational changes upon binding, in this case it indicates that the conformational equilibrium coupled to binding is not modulated by temperature within the experimental temperature range employed, and that there is no pre-existing equilibrium between the conformational states linked by zinc binding; in other words, the populations of the two conformational states linked by zinc binding (partially unfolded protein and folded protein) do not change significantly over the experimental temperature range in the absence of zinc. This has been termed “strong coupling” between binding and conformational change [15,40]. On the contrary, “weak coupling” occurs when the conformational equilibrium coupled to binding is modulated by temperature, in the absence of ligand within the experimental temperature range employed, and there is a pre-existing equilibrium between the conformational states linked by ligand binding; that is, the populations of the two conformational states linked by ligand binding change significantly over the experimental temperature range in the absence of ligand. Considering the distinction based on whether or not there is a pre-existing equilibrium between the ligand-free and ligand-bound protein conformational states, strong coupling can be identified with induced fit binding, whereas weak coupling is related to conformational selection.

**Figure 4:**
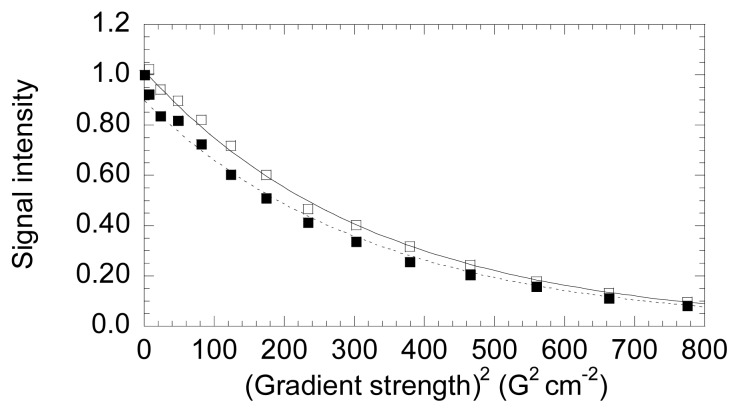
DOSY-NMR measurements to NS3. The exponential decay curves of NS3 in the absence (blank squares, continuous line) and in the presence of zinc (filled squares, dotted line). The units on the *y*-axis are normalized intensity of the up-field shifted signals. Experiments were acquired at 25 °C, at pH 5.4 (uncorrected for isotope effects). The errors in the intensities are less than 5%, as judged from the intensity of the signal-to-noise ratio in regions of the spectra where no signals are present. The intensities for both proteins were measured by integrating in 1D-NMR experiments the most up-field shifted resonances (see Appendix).

DOSY measurements were conducted to determine the hydrodynamic radii of both NS3 species and to compare with those obtained from theoretical calculations (Figure 4). Our measurements yielded translational diffusion coefficients of (8.1 ± 0.1) × 10^−7^ cm^2^·s^−1^ (for the two independent experiments the values were: (8.2 ± 0.2) × 10^−7^ cm^2^·s^−1^ and (8.0 ± 0.1) × 10^−7^ cm^2^·s^−1^; the stacked plots of these two independent measurements with two samples are shown in Figure S1) and (7.6 ± 0.2) × 10^−7^ cm^2^·s^−1^ (for the two independent experiments the values were: (7.7 ± 0.2) × 10^−7^ cm^2^·s^−1^ and (7.4 ± 0.2) × 10^−7^ cm^2^·s^−1^) for the zinc-bound and free NS3 species, respectively. In our conditions, the measured translational coefficient of dioxane was: 8.08 × 10^−6^ cm^2^ with a radius of 2.12 Å [41–43]. These data lead to hydrodynamic radii of 22.5 ± 0.4 and 21.1 ± 0.3 Å for the zinc-free and bound forms of NS3, respectively. Then, the zinc-free form has a slightly larger hydrodynamic radius. We can compare these values to that obtained from theoretical measurements. For a protein, the theoretical hydrodynamic radius can be calculated *ab initio* considering that the anhydrous molecular volume (MV̄/N ) equals the volume of a sphere: ${\text{r}}_{0}=\sqrt[{3}]{\frac{3\text{M}\bar{\text{V}}}{4\text{N}\pi }}$, where M is the molecular weight of the protein, V̄ is the partial specific volume of the protein, and N is Avogadro’s number. By taking the value of 0.70 cm^3^·g^−1^ for the average partial specific volume of a protein, the theoretical hydrodynamic radius is 18 Å. However, to allow comparison with the radii determined from DOSY, we should add to the calculated values a correction of 0.3 nm to account for the hydration shell [44]. Then the theoretical hydrodynamic radius of hydrated NS3 is 21 Å, close to the experimental value obtained for the zinc-bound form (21.1 Å). We estimated the volume of the zinc-bound protein (PDB number 1BT7) by using HYDROPRO [45], and we obtained a radius of 23.4 Å; the predicted translational diffusion coefficient was 8.46 × 10^−7^ cm^2^·s^−1^, close to the value determined experimentally.

It is important to indicate that the average increase in the radius of a particular unfolded state compared with that of the folded state is no larger than 15% when attaining a molten globule conformation [46]. For example, the addition of zinc to prothymosin α does not induce a complete folding of the protein, decreasing only slightly the radius of the resulting folded conformation (see [47] and references therein). Then, it seems that the zinc atom may act as a pin keeping the NS3 protease from opening, and the disruption of the NS3-zinc interaction leads to unfolding at certain extent in the molecule. However, the unstructured state is not a random coil and resembles a molten globule. The conformational change will move apart the catalytic residues, since H57 and D81 are located in the *N*-terminal domain, and S139 in the *C*-terminal domain, thus leading to enzyme inactivation. Therefore, in the absence of zinc, NS3 protease is mostly unstructured and can be considered an intrinsically (partially) disordered protein that folds upon binding to its zinc cofactor.

Atomic force microscopy (AFM) is the only technique presently available to obtain single-molecule images at sub-nm resolution in aqueous media [48]. Today, AFM may provide data on the topology, adhesion, elasticity, dynamics and other properties of biomolecules samples in physiologically relevant buffers [49]. However, only a few studies on protein morphology or protein association processes have been reported [50–57]. Here the direct visualization of single NS3 molecules allows characterizing the different molecular morphology related to the folding state involving zinc (see Appendix). Although there are different analyses devoted to the study of the folding pathways by force spectroscopy with AFM [58], morphological analysis of proteins in different folding states by using AFM imaging at the single molecule level are not found in the literature.

A clear series of AFM topography images of NS3 in the presence and absence of zinc were obtained, allowing the analysis of the impact of zinc on the morphology of NS3 molecules. The molecules were immobilized on an inert mica surface where previously enzymatic activities on protein samples were evaluated and observed they preserve the enzymatic functionality [57]. The protein was immobilized electrostatically on the negatively charged mica surface. NS3 presents a net positive charge at the working pH conditions due to its isoelectric point of 9.0. The concentration of the protein incubated on the mica sheets was suitable to get isolated features that could be analyzed individually. It is important to emphasize that the accuracy of the Z-height data reaches a sub-nm resolution thanks to the piezoelectric scanners. This does not happen in the *X*–*Y* plane, where the scanned features suffer the well documented tip broadening effect related to the AFM tip dilation that arise in higher sizes [59]. This effect does not affect the comparative analysis of the width related to the size or the association state of the protein molecules due to proportionality.

**Figure 5:**
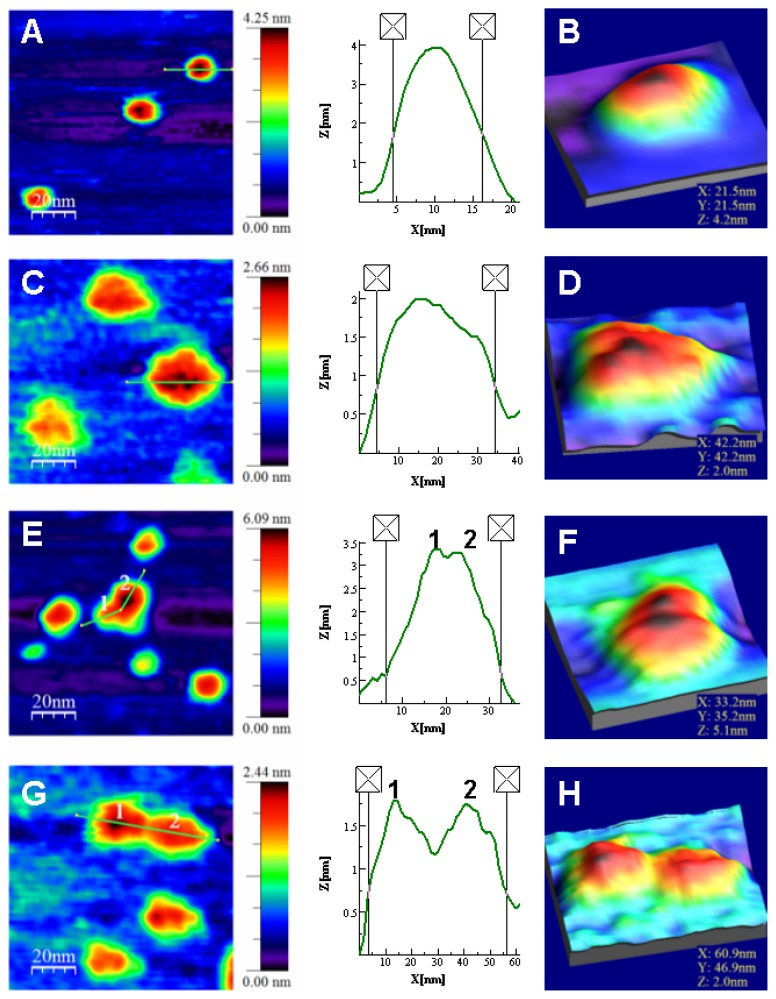
Topography TM-AFM images and *Z*-height profiles associated to the lines on selected features (any individually identified element in the images) of NS3 samples. (**A**) NS3 monomers in presence of zinc and *Z*-height profile on a single monomer amplified in (**B**) using the zoom WSXM function [60]; (**C**) NS3 monomers in the absence of zinc and *Z*-height profile on a single monomer amplified in (**D**); (**E**) Monomers and dimer found in zinc containing samples; *Z*-height profile associated to the dimer and (**F**) image of the dimer showed in detail; (**G**) Monomers and dimer found in samples with no zinc; *Z*-height profile associated to the dimer and (**H**) image of the dimer shown in detail. Numbers 1 and 2 indicate the corresponding monomers composing the dimers. The 2D images show scanned areas of 100 × 100 nm, meanwhile the areas of the 3D images were chosen to show the isolated feature in detail.

**Figure 6:**
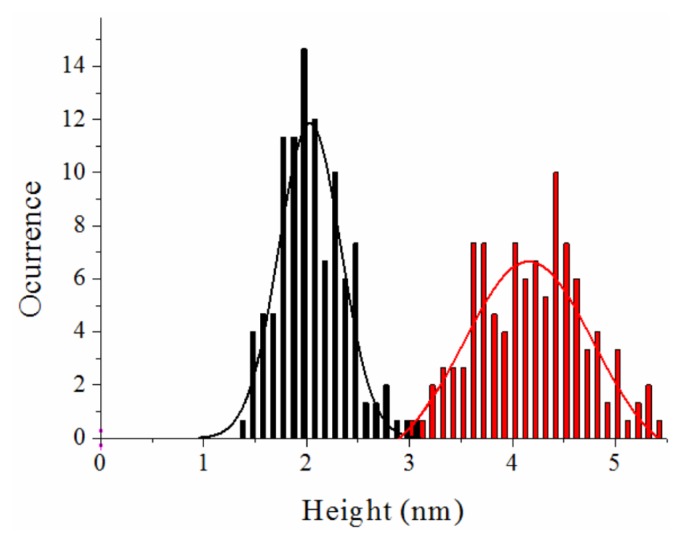
Height distributions of the NS3 molecules obtained from *Z*-height profiles on single monomers from TM-AFM images. The data are fitted to a Gaussian model. There is a peak centred in 4.2 ± 0.5 nm in the sample containing zinc (red bars). The sample with no zinc showed a peak centred in 2.0 ± 0.3 nm (black bars). The error can be attributed to the sub-nm accuracy of the technique in fluid and the different orientations of the molecules on the mica surface.

The results show that the structure of the NS3 protein is strongly zinc-dependent. The protein is mainly monomeric, showing an average height of 4.2 ± 0.5 nm in the presence of zinc ions (Figures 5A,B and 6). This size is only slightly higher than the exhibited by the structure deposited in the pdb due to hydration (code 1A1Q) [6]. In the analysis of protein association, 13% of the molecules were found to form dimers (Figure 5E,F) and only a marginal 3% associated as trimers (not shown). When protein was incubated without zinc, the analysis gave an average *Z*-height of about 2.0 ± 0.2 nm (Figures 5C,D and 6). When zinc ions were sequestered by EDTA, the molecules not only showed half the height to that of the above case, but a much less compact structure. This effect is easily observed in images showing isolated monomers in the presence (Figure 5A,B) or absence of zinc (Figure 5C,D) where the profiles associated to representative features show widths of around 12 and 30 nm, respectively. Though the size in the plane of AFM features appears dilated, as explained above, zinc-free protein exhibits around 2.5 times the size of the zinc-bound protein. This result suggests clearly a loss of structure and compactness in the molecules upon zinc dissociation. It is important to note that the size of the Zn-free NS3 species is larger than that measured by DOSY-NMR, which could be due to the fact that NMR is reporting averaged parameters overall the species present in solution and we are integrating the signals corresponding to the most up-field shifted species present in solution (where severe overlapping occurs), or that immobilization promotes partial unfolding of the protein.

The oligomerization pattern also changes, where only 4% of the zinc-free protein molecules form dimers. The morphology of the dimers in both type of samples also show the same trend. The profile of the dimer with zinc (Figure 5E) shows a *Z*-height of 3.4 nm and an *X*–*Y* width of around 26 nm, while that of dimer without zinc (Figure 5G) shows a *Z*-height of 1.8 nm and a *X*–*Y* width of around 52 nm. Although the *X*–*Y* width is broadened, it is possible to compare and conclude that monomers and dimers are much less compact in the zinc-free samples.

Therefore, these observations suggest a strong zinc dependence of the NS3 protease structure. The topology images show that the protein molecules exhibit a much expanded structure in absence of zinc, whereas in the presence of zinc the protein presents a much more compact properly folded structure.

In summary, all the experimental evidence obtained employing different biophysical techniques indicate the NS3-zinc interaction may be considered as a “folding by binding” event, a special limit case of allosteric interactions.

## 4. NS3 Protease: Structural Stability

Ligand binding often induces protein stabilization against unfolding if the ligand binds, at least preferentially, to the native state. The extent of the stabilization depends on the affinity of the interaction and the actual concentrations of protein and ligand. This is the general principle underlying allosteric control of proteins: a set of energetically, dynamically, structurally and functionally distinguishable protein states interacting specifically with a ligand, and that interaction shuffling the distribution of populations among those states (biochemical output) according to the concentration of ligand (biochemical input). Considering that the NS3 protease interacts with a zinc atom, stabilization of the protein against unfolding is expected in the presence of zinc.

**Figure 7:**
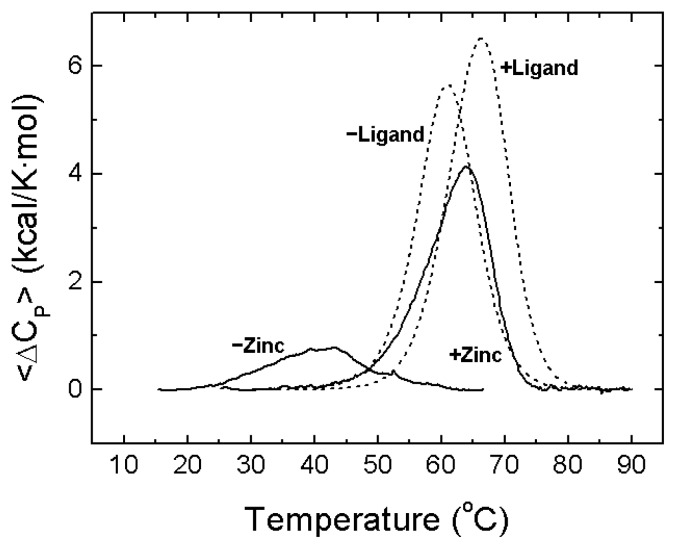
Structural stability of NS3 protease and modulation by zinc. Thermal denaturation scans of NS3 protease followed by differential scanning calorimetry (DSC) at pH 5 (continuous lines). Excess molar heat capacity is represented as a function of temperature. Protein concentration was 40 μM, and total zinc concentration was 0 and 40 μM. The NS3 protease exhibits the following thermal stability parameters: (mid-transition temperature of 30 °C, unfolding enthalpy of 18 kcal/mol, unfolding heat capacity of 1.2 kcal/K·mol). For comparison, simulated thermal denaturation scans (see Appendix) for a protein with similar molecular mass (mid-transition temperature of 60 °C, unfolding enthalpy of 70 kcal/mol, unfolding heat capacity of 2 kcal/K·mol) exhibiting a small conformational change coupled to ligand binding, in the absence and the presence of the ligand with similar affinity to that of zinc for NS3 protease (dissociation constant of 0.5 μM), are shown (dashed lines). Although the binding affinities for zinc and that hypothetical ligand are the same for their respective binding proteins, the extent and the magnitude of the stabilization are considerably different.

Calorimetric experiments have shown that zinc binding induces a strong stabilization of NS3 protease against thermal denaturation (Figure 7) and these results have also been confirmed by spectroscopic determinations [16]. Besides, chemical denaturations also support that conclusion. The extent of the stabilization depends on the concentration of free zinc and is in agreement with the binding affinity determined directly by ITC. The zinc-free protease shows very little stabilization energy; 0.4 kcal/mol at 20 °C (Figure 7), which leads to 34% of unfolded protein and 66% of unstructured-native protein at that temperature. At an equimolar concentration of zinc, the stabilization energy amounts to 1.9 kcal/mol, corresponding to 94% of folded-zinc-bound protein, 3% of unfolded protein and 3% of unstructured-native protein [16]. From these results, it is apparent that most (80%) of the structural stabilization energy of the NS3 protease is provided by the binding of the zinc cofactor (Figure 7) [16]. At higher zinc concentrations the stabilization energy and the percentage of fully folded protein increase accordingly. Because unfolding thermal and chemical transitions are observed for zinc-free protease, that conformational state is not equivalent to the fully unfolded protein and must have some residual structure. Other homologous zinc-dependent proteases do not show an unfolding transition in the absence of zinc, an indication of complete loss of structure upon zinc removal [61].

Because zinc binding induces a significant conformational change, as evidenced by calorimetric and spectroscopic techniques [15,16], the stabilization and the structural rearrangement extent and magnitude may be greater than those observed for a completely folded protein that binds a ligand with similar affinity but accompanied with a small conformational change. Usually, specific metal binding to a protein is accompanied by a considerable structural reorganization in that protein; the conformational change may be even larger than that elicited by the binding of a larger ligand. Then, the binding heat capacity (per ligand volume or surface area unit) is very large, compared to that of the binding of small organic ligands. Thus, specific metal ion binding to proteins is one of the key mechanisms for controlling protein conformation and function. In order to compare the zinc-induced stabilization in NS3 protease with that observed for ligand-induced stabilization in a completely structured protein, thermal denaturations have been simulated for a structured protein in the absence and the presence of a ligand with an interaction affinity similar to that of zinc binding to NS3 protease (Figure 7). In NS3 protease, zinc binding is accompanied by a large structural rearrangement (reflected in a large binding heat capacity) and the protein–ligand complex shows a markedly different stability compared to the free protein. Ligand binding to the structured protein elicits a small (if any) conformational change (reflected in a small binding heat capacity) and the stability increase is significantly smaller.

## 5. NS3 Protease: Conformational Landscape

NS3 protease is a small (20 kDa) globular monomeric protein, but exhibits a non-trivial behaviour and a complex conformational landscape. NS3 protease has two allosteric effectors: NS4A and zinc. The activation effects of both NS4A and zinc are associated to concomitant conformational changes elicited by their binding, enhancing the catalytic efficiency on the substrate. While NS3 protease presents some basal level of proteolytic activity in the absence of NS4A, it demonstrates no activity in the absence of zinc. Therefore, the conformational change induced by zinc must be larger than that induced by NS4A (there is crystallographic evidence about the latter, but none at all about the former). However, biophysical techniques provide information about the energetics and the structural changes associated with the effector binding. The binding of NS4A occurs at the *N*-terminal domain and induces structural rearrangements on the whole *N*-terminal domain and the configuration of the catalytic triad. In addition, the binding of NS4A affects the zinc binding site and, therefore, there exist a cooperative interaction in the binding of both NS4A and zinc ion [14]. If zinc is not bound, most of the protein structure is lost and the residual structure shows low thermodynamic stability; therefore, binding of NS4A to the zinc-free protease would have a considerable energetic penalty.

**Figure 8:**
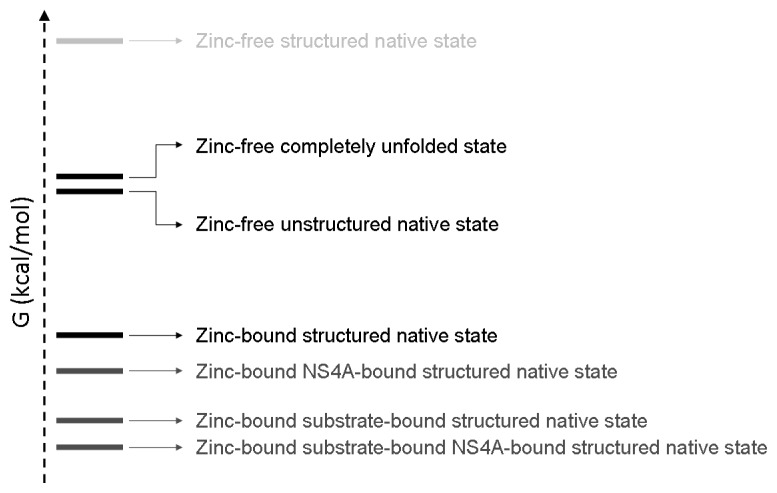
Schematic depiction of the conformational landscape of NS3 protease at 20 °C considering its intrinsic structural stability and its interactions with its different ligands: zinc, NS4A and substrate. States are populated according to their Gibbs energy. Ligand (zinc, NS4A, substrate) binding modulates and shifts populations depending on the ligand binding affinity (and free ligand concentration, also). In the absence of zinc, the energetic gap between the fully unfolded state and the unstructured native state is very small (~0.4 kcal/mol), and the fully structured native state is hardly populated (high Gibbs energy). Binding of zinc, NS4A and substrate reduces the Gibbs energy of the protein. Because the binding affinity of the substrate is larger than that of NS4A ([25,62] and unpublished data), the binding of substrate stabilizes (lowers the Gibbs energy and increases the population) NS3 protease to a larger extent. States with very low population due to energetic penalty, such as the zinc-free NS4A-bound protease, are not shown.

From the set of experiments reported (ITC, DSC, spectroscopy, NMR, AFM), it is apparent that the dissociation of zinc elicits a global conformational change in NS3 protease accompanied by a significant loss of structure. In addition, the zinc-free conformation maintains some residual structure. Therefore, regarding the zinc-protease interaction, at least three structurally, energetically and functionally distinguishable states must be considered: completely unfolded protein, zinc-free native partially-unstructured protein, and zinc-bound native structured protein. Taking substrate, NS4A and zinc into consideration, more than three conformational states must be considered, but some of them are hardly populated due to energetic penalties. Thus, the conformational landscape of NS3 is fairly intricate (Figure 8), including at least six different conformational states: completely unfolded protein, zinc-free native partially-unstructured protein, zinc-bound native structured protein, NS4A-zinc-bound protein, zinc-substrate-bound protein, and NS4A-zinc-substrate-bound protein, if the NS4A cofactor and the substrate are included as interacting partners. Other conformational states, such as NS4A-bound zinc-free protease, are possible, but less important (probable), because of their low population due to energetic penalty.

## 6. Conclusions

Protein ligand binding is often coupled with conformational changes. The extent of a ligand-induced conformational change depends on the intrinsic plasticity of the protein (that is, the population distribution over the ensemble of accessible conformational states) and the chemical structure and interaction affinity of the ligand. Therefore, in some proteins ligand binding hardly modifies the protein conformation (although it still may alter the protein dynamics), whereas in other proteins ligand binding triggers a considerable rearrangement of the protein conformation. This is more evident in intrinsically disordered proteins, because they exhibit an unstructured conformation, only adopting a well-defined conformation when bound to a given partner.

The different conformational states populated by a protein under some conditions can be classified as active or inactive, regarding a certain protein function. There are two basic strategies to block its activity: (1) targeting the active conformations with a competitive inhibitor of the substrate binding; and (2) targeting the inactive alternative conformations with a non-competitive allosteric inhibitor. Thus, in the first case the inhibitor enhances the stability of the active conformations, and shifts the conformational equilibrium towards those conformations, rendering the protein inactive by displacing and preventing the substrate interaction at the binding site. However, in the second case, the inhibitor enhances the stability of the inactive conformations, and shifts the equilibrium towards those conformations, rendering the protein inactive by trapping the protein into inactive conformations and reducing the population of the substrate binding-competent active conformations. In general, the broader the conformational landscape of protein, the bigger the number of inactive conformational states, increasing the number of potential targets for allosteric inhibition.

NS3 protease is a partially unstructured protein that requires a zinc atom in order to fold into an active conformation. Furthermore, NS3 requires the binding of the viral protein NS4A for an additional activity-enhancing conformational change and for proper intracellular membrane localization. Both cofactors are tightly regulated intracellularly. In particular, zinc, which is the main responsible for the structural stabilization of the active conformation of this enzyme, presents a very limited and kinetically-controlled intracellular availability. Therefore, NS3 folding into the active conformation occurs in very unfavorable environment, which might help to design strategies for allosteric inhibitors targeting alternative non-native conformations. This approach can be implemented for other protein targets exhibiting a structure strongly dependent on scarce cofactors (e.g., structural metal ions), and a comprehensive (structural and functional) biophysical characterization of the protein target will provide the necessary information.

## Figures and Tables

**Figure A1:**
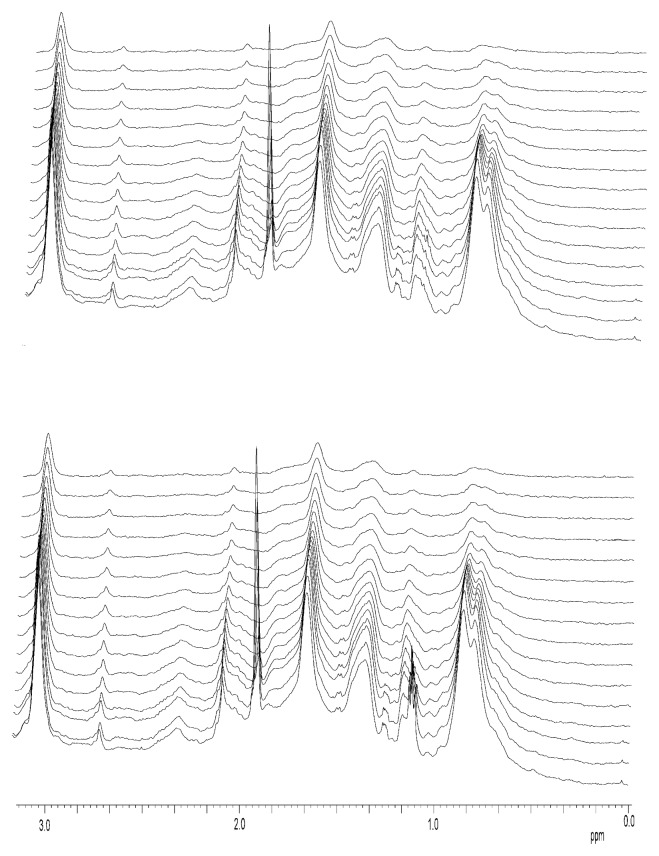
Stacked plots for DOSY-NMR measurements to zinc-bound NS3 protease. The exponential decay curves of NS3 shown in Figure 4 of the main text were obtained from 1D-plots as those shown in this figure, by integration of the peaks between 1.0 and 0.5 ppm. The amide region did not show any peak, apart of the aromatic residues, due to the fact that DOSY experiments were carried out in D_2_O, after several hours of exchange in Amicon centrifugal devices. The two different stacked plots for the two different samples of the zinc-bound NS3 are shown (see main text for the diffusion coefficients obtained). For the zinc-free NS3 similar plots were obtained. Experiments were acquired at 25 °C, at pH 5.4 (uncorrected for isotope effects).

## Appendix

### Protein Expression and Purification

NS3 protease from HCV genotype 1b J-strain is encoded in a pET7 plasmid (a kind gift from Dr. C. Steinkühler, Istituto di Ricerche Molecolare, P.Angeletti, Rome). The 180-residue protease sequence contains a solubilizing *C*-terminal tail (-SKKKK). Transformed BL21 Star (DE3) One Shot cells (Invitrogen, Barcelona, Spain) were grown in LB medium supplemented with ampicillin at 37 °C. Induction of protein expression was initiated when optical density at 600 nm reached 0.6–0.8 by adding 0.4 mM IPTG and zinc chloride 0.1 mM, and progressed for 3–5 h at 23 °C. Cells were collected and resuspended in sodium phosphate 5 mM pH 7.5, NaCl 150 mM. Then cells were collected again, and resuspended in Buffer A (Tris HCl 10 mM, DTT 5 mM, glycerol 10%, zinc chloride 100 μM) and broken by sonication. The supernatant was passed through a Q-Sepharose anion exchange column followed by an SP-Sepharose cation exchange column, in an ÄKTA FPLC System (GE Healthcare, Barcelona, Spain) at a 5 mL/min flow (with the absorbance monitored at 280 nm). Protein bound to the SP-Sepharose resin was eluted using two ionic strength gradients (0–150 mM and 150–500 mM sodium chloride) with Buffer A and sodium chloride 1 M added. For storage, protein was dialyzed in buffer MOPS 10 mM pH 7, zinc chloride 10 μM. For sample preparation, a rapid buffer exchange process to sodium acetate 100 mM, pH 5, zinc sulphate 50 μM, was made, using Amicon centrifugal devices (Millipore, Barcelona, Spain). Considering the affinity of NS3 for zinc [15], the final concentration of free zinc after several concentration-dilution steps by centrifugation must be close to 50 μM.

The apo-NS3 was obtained as described [15]. Briefly, purified NS3 was concentrated and diluted at least ten times in Amicon centrifugal devices in the presence of 2 mM EDTA at pH 5 (sodium acetate 100 mM). Because at pH 5 the affinity of EDTA for zinc binding is three orders of magnitude larger than that of NS3 for zinc binding at 15–25 °C (and presumably at other temperatures, according to the heat capacities and enthalpies determined for zinc binding to EDTA and NS3 protease), all zinc ions were removed from the protein [15]. Experimental evidences (NMR, fluorescence and CD spectroscopies, and ITC) indicated that zinc-free NS3 protease loses most of its tertiary structure, but retains most of its secondary one [15].

### Nuclear Magnetic Resonance Spectroscopy

^1^H NMR experiments were carried out in a Bruker Avance DRX-500 with gradients along the *z*-axis. All experiments were performed in a TXI probe at pH 5.4 in deuterated acetic buffer (100 mM) (Sigma, Madrid, Spain) in the absence or in the presence of SO_4_Zn at a final concentration of 1 mM in D_2_O. The measured pH was not corrected for isotope effects. Experiments were acquired at 25 °C. Protein concentration was in all cases ~140 μM. Two independent measurements were carried out with two different samples for each protein species.

Translational self-diffusion measurements (DOSY experiments) were performed using the pulsed-gradient spin-echo NMR method. The following relationship exists between the translational self-diffusion parameter, D, and the NMR parameters [63,64]:

(A1) $$\frac{\text{I}}{{\text{I}}_{0}}=-\text{exp}\left(\text{D}\gamma_{\text{H}}^{2}\delta^{2}{\text{G}}^{2}\left(\Delta -\frac{\delta }{3}-\frac{\tau }{2}\right)\right)$$

where *I* is the measured peak intensity (or volume) of a particular (or a group of) resonance(s); *I*_0_ is the maximum peak intensity of the same (group of) resonance(s) at the smaller gradient strength; *D* is the translational self-diffusion constant (in cm^2^ s^−1^); δ is the duration (in s) of the gradient; *G* is the strength of the gradient (in T cm^−1^); Δ is the time (in s) between the two gradients (*i.e.*, the time when the molecule evolves); and τ is the time (100 μs) for gradient recovery at the end of each scan. Data can be plotted as −ln(*I*/*I*_0_) *vs. G*^2^, and the slope of the line is $\text{D}\gamma_{\text{H}}^{2}\delta^{2}{\text{G}}^{2}\left(\Delta -\frac{\delta }{3}-\frac{\tau }{2}\right)$, from which *D* can be easily obtained. Determination of the hydrodynamic radii of the protein under both conditions was carried out in the presence of 0.1% of dioxane as described by Dobson and co-workers, and others [41–43]. The radius of dioxane was 2.12 Å. Examples of the stacked plots for the two independent measurements with two samples are shown in Figure A1.

The gradient strength was calibrated using the diffusion rate for the residual proton water line in a sample containing 100% D_2_O in a 5-mm tube, and back-calculating *G*. This procedure assumes that the diffusion rate for HDO in a 100% D_2_O sample is 1.94 × 10^−5^ cm^2^ s^−1^ at 25 °C [65]. Experiments were acquired by using the longitudinal eddy current delay pulse sequence, with a post gradient eddy-current relaxation delay of 5 ms. Each experiment was averaged over 1 K scans and the number of data points was 16 K. The strength of the gradients was varied from 2% of the total power of the gradient coil to 95%, and their shape was a sine function. For experiments with NS3, the duration of the gradient was varied between 2.9 (without zinc) and 2.8 ms (with zinc), and the time between both gradients was 175 ms. The most up-field shifted methyl groups (between 0.5 and 1.0 ppm) were used to measure the changes in intensity of the DOSY signals. Errors in the intensities were estimated to be 5%, as judged from the integration of spectral regions where no signal was present.

### Atomic Force Microscopy Imaging

Measurements were performed using a MultiMode 8 AFM system (Bruker Digital instruments, Santa Barbara, CA, USA). The operation mode used was Tapping Mode (TM) [66]. In TM image operation the cantilever driven by a piezoelectric actuator vibrates at its resonance frequency. Upon approaching the sample, the tip briefly touches the surface at the bottom of each swing, resulting in a decrease in oscillation amplitude. By maintaining constant oscillation amplitude, the image of the surface is obtained allowing high resolution images of easily damaged samples. V-shaped silicon nitride cantilevers with integrated pyramidal tips with spring constants of 0.01 N/m to 0.03 N/m and nominal frequencies of 7–15 kHz (Bruker Probes, MSNL lever Probes) were employed. Cantilevers were calibrated using the thermal noise method [67]. The main requirement is the necessity of immobilizing the sample on a nanoflat surface in order not to be swept away by scanning.

All experiments used 1 μM NS3 and were conducted in a buffered environment with sodium acetate 8.3 mM, pH 5 at room temperature. Zinc at concentration of 0.8 mM zinc or no zinc (EDTA incorporated) was added and mixed for 30 min under mild stirring. Protein samples were incubated on small fresh exfoliated muscovite mica pieces (Electron Microscopy Sciences, Hatfield, UK) for 10 min at room temperature and washed three times to remove the excess of non-immobilized protein. The sample and holder were introduced in a liquid cell previously washed with isopropanol 20% and Millipore ultrapure water. The protein concentration used allowed visualizing individual features. Raw images were processed using the WSxM freeware [60]. A considerable amount of features were analyzed one to one for the statistical population study of the different heights and types of association of the NS3 protein. Only clearly identified features were quantified discarding unclear elements [55]. *Z*-height profiles were applied on the features to calculate the size in *Z* with sub-nm accuracy. To estimate the average in size of the molecules a Gaussian distribution of at least 150 features was applied.

### Differential Scanning Calorimetry

DSC assays were performed as reported previously [16]. The heat capacity of NS3 as a function of temperature was measured with a high precision differential scanning VP-DSC microcalorimeter (MicroCal, Northampton, MA, USA). Protein samples and reference solutions were properly degassed and carefully loaded into the cells to avoid bubble formation. Thermal denaturation scans were performed with freshly prepared buffer-exchanged protein solutions. The baseline of the instrument was routinely recorded before the experiments. Experiments were performed in 100 mM sodium acetate, pH 5, at a scanning rate of 1 °C/min. Experiments were carried out with 40 μM of protein and different total concentrations of zinc. Different concentrations of zinc were achieved by adding zinc or EDTA from calibrated reference stock solutions. Thermal denaturations were reversible (as judged by the agreement of the thermogram shapes and sizes between the first and the second scans), concentration independent (with regard to the protein), and scan-rate independent. Pre- and post-transition baselines were considered linear with temperature.

Intrinsic protein stability parameters were obtained by analyzing thermal scans for ligand-free protein considering a two-state unfolding model. When a ligand is present, whether at equal or higher concentration than that of the protein, and irrespective of the ligand binding affinity, the system must be considered as constituted by the protein, with intrinsic stability parameters equal to those of the ligand-free protein, and the ligand, with intrinsic binding parameters, modulating the protein stabilization energy. Thus, any perturbation in the system will involve the explicit coupling between two equilibria: conformational equilibrium and ligand binding equilibrium. The influence of the ligand on the conformational stability of the protein will be reflected through that coupling.

The average enthalpy of the system, <Δ*H*>, is given by:

(A2) $$\langle \Delta \text{H}\rangle ={\text{F}}_{\text{U}}\Delta \text{H}={\text{F}}_{\text{U}}\left(\Delta {\text{H}}_{0}-{\text{F}}_{\text{B}}\Delta {\text{H}}_{\text{B}}\right)={\text{F}}_{\text{U}}\left(\Delta {\text{H}}_{0}-\langle \Delta {\text{H}}_{\text{B}}\rangle \right)$$

where Δ*H*_0_ is the intrinsic unfolding enthalpy, Δ*H*_B_ is the binding enthalpy, *F*_U_ is the fraction of unfolded protein, *F*_B_ is the fraction of protein bound to ligand. Then, the excess heat capacity, <Δ*C*_P_>, is calculated as the temperature derivative of the enthalpy:

(A3) $$\langle \Delta {\text{C}}_{\text{P}}\rangle =\frac{\partial \langle \Delta \text{H}\rangle }{\partial \text{T}}$$

If we are interested in the parameters for the unfolded state, we get for the Gibbs energy, the unfolding enthalpy and the unfolding heat capacity:

(A4) $$\begin{array}{l} \Delta \text{G}=\Delta {\text{G}}_{0}+\text{RT ln}(1+{\text{K}}_{\text{B}}[\text{L}])=\Delta {\text{G}}_{0}-\langle \Delta {\text{G}}_{\text{B}}\rangle \\ \Delta \text{H}=\Delta {\text{H}}_{0}-{\text{F}}_{\text{B}}\Delta {\text{H}}_{\text{B}}=\Delta {\text{H}}_{0}-\langle \Delta {\text{H}}_{\text{B}}\rangle \\ \Delta {\text{C}}_{\text{P}}=\Delta {\text{C}}_{\text{P}0}-{\text{F}}_{\text{B}}\Delta {\text{C}}_{\text{PB}}-{\text{F}}_{\text{B}}(1-{\text{F}}_{\text{B}})\frac{\Delta {\text{H}}_{\text{B}}^{2}}{{\text{RT}}^{2}}=\Delta {\text{C}}_{\text{P}0}-\langle \Delta {\text{C}}_{\text{PB}}\rangle \end{array}$$

which are valid in the case of ligand concentration much higher than that of the protein, where ΔG_0_ is the intrinsic unfolding Gibbs energy, Δ*C*_P0_ is the intrinsic unfolding heat capacity, *K*_B_ is the ligand association constant, Δ*G*_B_ is the intrinsic binding Gibbs energy, Δ*H**_B_* is the intrinsic binding enthalpy, F_B_ is the fraction of ligand-bound protein, Δ*C*_PB_ is the intrinsic binding heat capacity, and *R* is the ideal gas constant. It is clear from the previous equations that the apparent unfolding parameters are a function of the intrinsic unfolding parameters and the intrinsic ligand binding parameters, and the presence of the ligand modulates the apparent unfolding parameters. All the intrinsic parameters (for conformational and binding equilibria) are temperature-dependent. In order to evaluate numerically the excess heat capacity (Equation 3), the fraction of protein in the different conformational states must be calculated. The mass conservation for each species provides a set of equations:

(A5) $$\begin{array}{l} {\left[\text{P}\right]}_{\text{T}}=[\text{N}]+[\text{NL}]+[\text{U}]=[\text{N}]+{\text{K}}_{\text{B}}[\text{N}][\text{L}]+{\text{K}}_{0}[\text{N}]\\ {\left[\text{L}\right]}_{\text{T}}=[\text{L}]+[\text{NL}]=[\text{L}]+{\text{K}}_{\text{B}}[\text{N}][\text{L}] \end{array}$$

which can be solved numerically or analytically. Here, *U* is the unfolded protein, *N* is the zinc-free native protein, and *NL* is the zinc-bound native protein. Once the free concentrations [N] and [L] are known, the concentration of each conformational/liganded state can be calculated. Finally, the excess heat capacity can be calculated at each experimental temperature from the previous equations. Data were analyzed/simulated using software developed in our laboratory implemented in Origin 7 (OriginLab, Northampton, MA, USA).
